# What Is the Bioscience Industry Doing to Address the Ethical Issues It Faces?

**DOI:** 10.1371/journal.pmed.0030142

**Published:** 2006-04-04

**Authors:** Carlos Novas

## Abstract

Novas discusses the implications of a new study in *PLoS Medicine,* in which executives and senior managers from bioscience firms were asked what their companies were doing to promote ethical behaviour.

Corporations are increasingly expected to behave ethically and to make their operations as transparent as possible. This is especially the case for the bioscience industry (biotechnology and pharmaceutical firms). These firms deal with a commodity like no other: our health and well-being. They also engage in research that manipulates the basic building blocks of life and challenges common understandings of the boundaries between humans and animals, the treatment of disease versus the enhancement of healthy lives [ 1], and when life begins or ends. To address these complex challenges, bioscience corporations have begun to implement a range of practices to address the scope of ethical questions that their operations pose. In a new Policy Forum article published in *PLoS Medicine*, Jocelyn Mackie and colleagues describe the variety of mechanisms that bioscience firms have put in place to address the ethical issues confronting them [ 2].

## The Methodology

Based on more than 100 interviews with executives and senior managers from 13 firms, the authors sought to find out what these professionals had to say about what their companies are doing to promote ethical behaviour. To date, there has been relatively little empirical research on this topic. As such, it is a timely piece.

The authors draw upon a larger study, in which detailed case studies were developed for each firm, including the ethical challenges that the firms faced and the mechanisms used to address them. The firms selected for analysis were chosen because they were known to be developing innovative approaches to dealing with ethical issues. They were further selected to represent the diversity of this industrial sector and to account for variations in firm size and location. In the paper, the authors seek to draw comparatively upon the case studies they developed to highlight the range and variety of mechanisms adopted by firms to address ethical issues.

## Approaches Used by Firms to Encourage Ethical Behaviour

Based on the analysis presented, firms of all sizes and in different market niches are using a variety of approaches to encourage ethical behaviour. First of all, executives are promoting ethics as part of a firm's core values. There is also evidence of specialisation: larger firms are able to create dedicated departments, while smaller firms are incorporating ethics into the responsibilities of senior managers.

Corporations are increasingly expected to behave ethically and to make their operations as transparent as possible.

The companies studied have further retooled their organisational structures with ethics in mind: ethics shaped their hiring and staff performance evaluations, employees in some firms were given ethics training, and visual and oral reminders were being used in the workplace to reinforce an organisation's commitment to its ethical values. In instances where internal expertise was lacking, external consultants were brought in or independent ethics advisory boards were created to provide guidance and advice. Regardless of size, most of the firms studied were engaging with a range of stakeholders—whether it be reshaping their relationships with suppliers to maintain high ethical standards, consulting with a local community, inviting activists to visit laboratory facilities, or launching corporate philanthropy programmes in Africa. Lastly, firms are beginning to develop measures for evaluating and reporting their ethical behaviour.

## The Implications

This study demonstrates that (at least for a selective range of firms in the bioscience industry) some corporations have started to believe that the types of relationships they have with patients, carers, families, physicians, activists, partners, suppliers, regulators, and the public are an essential element of corporate financial and social viability. Considering that some of the firms studied do not seem to have any products on the market, current and anticipated ethical concerns are shaping their corporate practices and bottom lines in the here and now. This paper also provides evidence of how ethical decision making is not an entirely abstract philosophical exercise: ethics has to be embedded in a range of social practices and relationships that need to be continually cultivated and reinforced if they are to be effective.

Lastly, it can be extrapolated that ethics is an asset that firms can trade upon. Firms are considering ethics as central not only to their research activities and the dissemination of their products to consumers, but also to the reputation and branding of the company itself. Of course, ethics, like any other asset, has multiple values. Ethics can be used by firms not only to shape their decisions, but to aid corporate public relations campaigns. No doubt, the multifaceted character of corporate ethics programmes can lead to outbreaks of scepticism amongst bioethicists and the public.

## Unresolved Questions

A question that was left unresolved by the authors was how effective these mechanisms have been at accomplishing their objectives. Whilst firms may not yet be evaluating the actual impact of these mechanisms, it is imperative for bioethicists studying these mechanisms to discuss their relative merits and disadvantages. Better yet would be an evaluation of how ethical these companies actually are. In a similar vein, we need to know more about how and to what extent the mechanisms adopted by firms influenced their research, investment, or marketing decisions. Finding out about such influence will require further empirical analysis and a willingness on the part of executives in the bioscience industry to let researchers not only interview them but also observe what goes on in their boardrooms. This type of transparency would facilitate the investigation of what kinds of problems do and do not get defined as being ethical and the organisational processes that shape firm behaviour. Lastly, it is important to emphasise that firms, like individuals, do not live in isolation. We need to pay greater attention to the broader political, economic, and social context that has encouraged firms to develop mechanisms to address ethical issues, as well as the role of industry organisations and professional associations in facilitating the uptake and dissemination of these mechanisms.

It will be important to study bioscience firms as they start to incorporate ethics into their organisational practices and into the very products they develop through their research decisions. To date, this subject area has not been extensively explored by bioethicists. As more aspects of our health and illness are embraced by the bioscience industry, the ethical issues surrounding industry's actions will become an area that is ripe for analysis.

## The Relationship between Ethicists and Corporations

A question that this paper opens up for further analysis and debate, given that firms are starting to integrate ethics into their organisational practices, is how and through what forms bioethicists should relate to corporations. At the present time, there seem to be two dominant forms. One form appeals to the values long cherished in academe: independence, critical scholarship, credibility, and integrity [ 3, 4]. The other form appeals to the values to be gained by consulting for industry: corporate or policy relevance, alongside honoraria, salaries, and grants [ 5]. Presently, bioethics seems to be at an impasse over what form to adopt.

Perhaps the lessons to be learned come from the bioscience industry itself. As firms have started to reformulate their organisational forms and modes of conduct in relation to changing socioeconomic circumstances, perhaps it is an opportune moment for bioethicists to rethink the subjects they choose to study and how bioethics engages with its various stakeholders. Just as scientists have created new theories and techniques to investigate the phenomena of life, bioethicists need to develop new concepts and tools for proposing how we should individually and collectively relate to one another in a manner that is capable of dealing with the dilemmas that will be posed by the provision of health in the 21st century.
